# Penetrating Wounds of Great Cavities

**Published:** 1874-08

**Authors:** James C. Rea


Penetrating Wound of the Abdomen and Chest.—*By James C. Rea, M.D.*—* * * *Remarks.*—These cases, apart from their intrinsic interest as examples of recovery from severe injuries, seem worthy of record as bearing upon the vexed question of the *constitutional treatment* to be adopted in the management of penetrating wounds of the great cavities. In the first place, it will be observed that in none of the cases was *blood-letting*, either general or local, found necessary. Secondly, what is called *absolute diet* was not prescribed in any instance, but on the contrary, the patients were encouraged from the very beginning to take as much milk as possible, and in the course of a few days were given beef-tea in addition, while in Case I. the use of alcoholic stimulus was resorted to at an early period. Thirdly, *opium* was freely administered in all the cases, combined at first with small doses of *calomel*, with a view of obtaining what may be called the anticipatory antiphlogistic effect of this remedy. Of course, had the bowel been wounded in Case I., mercury would not have been given, for fear of increasing the risk of fecal extravasation; but it is believed that this drug exercises a salutary influence upon the reparative process in wounds of serous membranes. *Quinia* and *iron* were given in large doses during convalesence in Case I. Finally, attention is invited to these cases as illustrations of the benefit to be derived from the *restorative method* in wounds of the thoracic and abdominal cavities—a method which it is believed is destined to win as much favor in surgical as it has already in medical practice.

